# Identification of White Matter Lesions in Patients With Acute Ischemic Lesions Using U-net

**DOI:** 10.3389/fneur.2020.01008

**Published:** 2020-09-30

**Authors:** Shuai Liu, Xiaomeng Wu, Shengji He, Xiaowei Song, Fei Shang, Xihai Zhao

**Affiliations:** ^1^Department of Biomedical Engineering, Center for Biomedical Imaging Research, School of Medicine, Tsinghua University, Beijing, China; ^2^Department of Biomedical Engineering, School of Life Science, Beijing Institute of Technology, Beijing, China; ^3^Department of Neurology, School of Clinical Medicine, Beijing Tsinghua Changgung Hospital, Tsinghua University, Beijing, China

**Keywords:** white matter lesions, acute ischemic lesions, lesion segmentation, FLAIR, DWI, U-net

## Abstract

**Background:** White matter lesions (WML) have been proved to be significantly associated with many brain diseases. Precise evaluation of burden of WML at early stage could provide insights in the prognosis and assist in intervention. However, acute ischemic lesions (AIL) exhibit hyperintensities on FLAIR images either, and are detected by diffusion weighted imaging (DWI). It is challenging to identify and segment WML in the patients with WML and AIL. Convolutional neural network (CNN) based architecture has been validated as an efficient tool for automatic segmentation. This study aimed to evaluate the performance of U-net in evaluation of WML in the patients with WML and AIL.

**Methods:** A total of 208 cases from Chinese Atherosclerosis Risk Evaluation (CARE II) study were recruited in the present study. All subjects underwent imaging of FLAIR and DWI on 3.0 Tesla scanners. The contours of WML delineated by the observer and its scores rated by the observer were considered as gold standard. Among all 208 cases, 108 were randomly selected as train set, and the remaining 100 cases were used as test set. The performance of lesion segmentation toolbox (LST) and three U-net models were evaluated on three levels: pixel, lesion, and subject levels. The performance of all methods in WML identification and segmentation was also evaluated among the cases with different lesion volumes and between the cases with and without AIL.

**Results:** All U-net models outperformed LST on pixel, lesion, and subject levels, while no differences were found among three U-net models. All segmentation methods performed best in the cases with WML volume (WMLV) > 20 ml but worst in those with WMLV < 5 ml. In addition, all methods showed similar performance between the cases with and without AIL. The scores determined by U-net exhibited a strong correlation with the gold standard (all Spearman correlation coefficients >0.89, ICCs >0.88, *p*-values <0.001).

**Conclusion:** U-net performs well on identification and segmentation of WML in the patients with WML and AIL. The performance of U-net is validated by a dataset of multicenter study. Our results indicate that U-net has an advantage in assessing the burden of WML in the patients suffered from both WML and AIL.

## Introduction

White matter lesion (WML), also known as white matter hyperintensities (WMH), is a type of cerebral small vessel disease which is highly prevalent in the elderly (>60 years old) (1, 2). Severity of WML will significantly increase with aging, and is associated with stroke (3, 4), depression (5), Alzheimer's disease (6), and migraine (7). At early stage of WML, the changes in tissue fluid mobility and water content are reversible. However, irreversible demyelination and axon injury will appear if intervention is not conducted (8). Therefore, evaluation of WML at early stage can provide insights in the prognosis and assist in the intervention.

Magnetic resonance imaging fluid attenuated inversion recovery (MRI-FLAIR) has been widely used in quantifying the burden of WML which exhibits hyperintensity on FLAIR images. However, some patients with WML always suffer from acute ischemic lesions (AIL) which is characterized by hyperintensity on FLAIR images as well. The similarity of intensity between WML and AIL on FLAIR images makes it difficult to distinguish between WML and AIL and thus evaluate the severity of WML in the patients with both WML and AIL. Therefore, quantification of WML in the patients with WML and AIL relies on the precise identification and segmentation of AIL. Diffusion weighted imaging (DWI) is a technique to detect AIL. Different from WML, AIL exhibits hyperintensity on DWI images. The combination of FLAIR and DWI imaging modalities might be a potential for improving the precision of WML identification and segmentation in the patients with both WML and AIL.

Originally, precise evaluation of WML always relies on manual delineation, which is laborious and tedious. Now, convolutional neural network (CNN), as a type of supervised learning, has been validated as an efficient tool for automatic segmentation on medical images (9–13). Many CNN-based methods were proposed to segment brain tissues as well as lesions. Moeskops et al. (14) reported that CNN-based method could accurately segment brain tissues *via* integrating T1W, T2W, and T1W inversion recovery (IR) images. Guerrero et al. (15) proposed a CNN architecture (uResNet) to segment and differentiate WMH and stroke lesions by combining T1 and FLAIR images. Woo et al. (16) compared CNN with conventional algorithms in segmenting AIL on DWI images. Duong et al. (17) adapted a 3D U-net architecture for automatic segmentation of lesions on FLAIR images. Winzeck et al. (18) accurately segmented AIL on multi-parametric DWI images using ensemble of CNN. Atlason et al. (19) trained the CNN in an unsupervised manner to automatically segment WMH.

However, seldom studies focused on the identification and segmentation of WML and AIL on FLAIR images. In the present study, we investigated the performance of U-net in identification and segmentation of WML in patients suffered from both WML and AIL. The performance of CNNs with the U-net architecture was compared in the identification and segmentation of WML: (a) using only FLAIR image as a single input; (b) using FLAIR image and DWI image as two single inputs, respectively; and (c) using the combination of FLAIR and DWI images as a two-channels input. This paper was organized as follows: section Materials presented the dataset, evaluation metrics and statistical analysis. Section Methods described the pre-processing, augmentation, U-net architectures and three models in detail. Section Results exhibited the identification results, and section Discussion interpreted the key results of the current study.

## Materials

### Dataset

A total of 208 subjects from a cross-sectional multicenter study of Chinese Atherosclerosis Risk Evaluation (CARE II) (20) were used in the present study, and all subjects suffered from WML. The aim of CARE II study was to investigate the prevalence of high-risk carotid atherosclerotic plaques in patients with recent symptoms (within 2 weeks). The study design had been detailed in previous publication (20). Every aspect of this study was approved by a local institutional review board and a signed consent was obtained from each subject. All subjects underwent FLAIR and DWI scan on 3.0 Tesla MR scanners with 8-channel phase array coils. A stack of 18–22 slices was acquired on transverse section. The imaging parameters of FLAIR and DWI sequences are exhibited in Table 1.

**Table 1:** Imaging parameters of FLAIR and DWI sequences.

	**FLAIR**	**DWI**
Sequence	TSE	EPI
Repeat time, ms	5,000–11,000	1,400–6,000
Echo time, ms	84–186	43–92
Inversion time, ms	2,000–2,800	/
Field of view, mm^2^	200 × 200–260 × 260	200 × 200–260 × 260
Matrix	256 × 256–512 × 512	192 × 192–288 × 288
Slice thickness, mm	5–7	5–7
Pixel spacing, mm	0.43–0.49	0.80–1.20

*TSE, turbo spin echo; EPI, echo planar imaging*.

### Gold Standard

**Figure 1:**
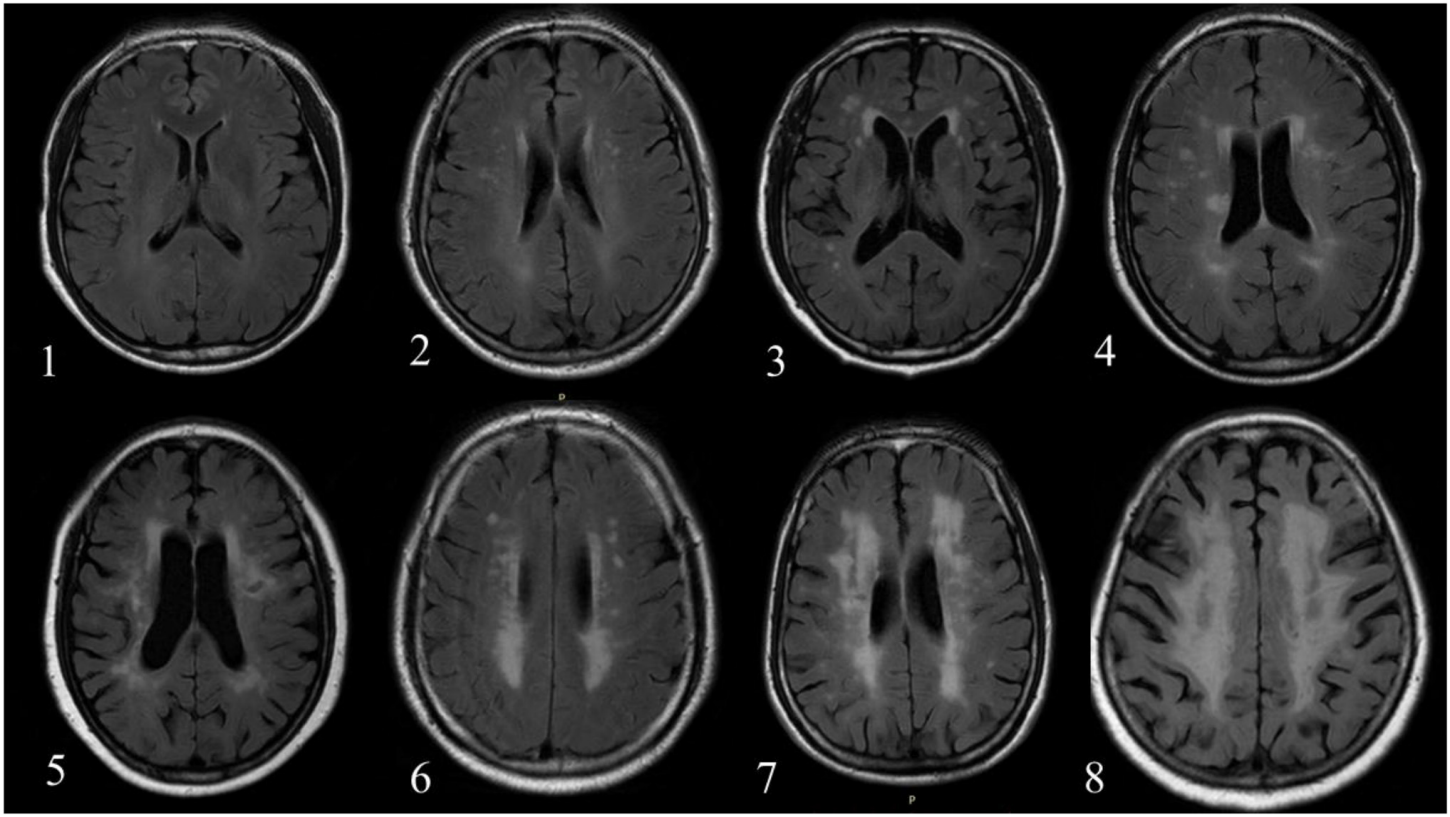
The number in left bottom represented the scores of WML determined by reviewers. No case with score 9 was found in the current dataset.

All MR images were reviewed by two radiologists with >5 years' experience in neuroradiology with consensus. The contours of WML and AIL manually delineated by the observers were considered as the gold standard. Subsequently, a rating scheme from 0 to 9 was used to evaluate the WML burden of each individual (21) (Figure 1). Table 2 exhibits the distributions of the WML rating scores in training and testing sets.

**Table 2:** The distribution of WML ratting scores and cases with AIL in training and testing sets.

**WML**	**Training set**		**Testing set**	
**rating score**	**(*****n*** **=** **108)**		**(*****n*** **=** **100)**	
	**Case number**	**Cases with AIL**	**Case number**	**Cases with AIL**
	**(*n* = 108)**	**(*n* = 52)**	**(*n* = 100)**	**(*n* = 46)**
0	0	0	1	1
1	28	15	29	10
2	36	15	30	14
3	21	11	19	13
4	3	2	3	1
5	6	4	8	2
6	4	1	5	3
7	9	4	5	2
8	1	0	0	0

### Evaluation Metrics

The results of segmentation were evaluated at three levels: pixel, lesion, and subject levels.

#### Evaluation at Pixel Level

The segmentation at pixel level was evaluated in each subject using dice similarity coefficient (DSC), Recall, and Precision as follows:

$$\begin{array}{r} DSC=\frac{2^{*}\left(S_{G}\cap S_{P}\right)}{S_{G}+S_{P}} \end{array}$$

$$\begin{array}{r} Recall=\frac{\;S_{G}\cap S_{P}}{S_{G}} \end{array}$$

$$\begin{array}{r} Precision=\frac{S_{G}\cap S_{P}}{S_{P}} \end{array}$$

where S_G_ represented the ground truth and S_P_ represented the segmentation results generated by U-net.

#### Evaluation at Lesion Level

True positive ratio (TPR) and false positive ratio (FPR) were calculated using the equations as follows:

$$\begin{array}{r} TPR=\frac{TP}{TP+FN} \end{array}$$

$$\begin{array}{r} FPR=\frac{FP}{FP+TN} \end{array}$$

where TP represented the number of the lesions detected, FN represented the number of the lesions undetected, FP was the number of the normal regions which were incorrectly detected as lesions, and TN was the number of the normal region correctly detected.

## Methods

### Pre-processing

Some CNN-based networks without pre- or post-processing had been proposed for automatic segmentation of brain lesions on MR images (22, 23). However, a pre-processing was still conducted in the present study to normalize all the slices and discard the slices without white matter, gray matter and cerebrospinal fluid (CSF). Firstly, all FLAIR and DWI images were normalized to a uniform size (256 × 256) by cropping or padding. Secondly, different structures of brain were automatically segmented using SPM toolbox without manual parameter tuning (https://www.fil.ion.ucl.ac.uk/spm/) (Figure 1), and the slices without white matter, gray matter and CSF were abandoned. Finally, a linear normalization was applied to normalizing the intensity of whole volume ([0, 255]).

### Data Augmentation

In neural networks, data augmentation could improve the robustness and precision of results *via* adding the cases with different transformations into dataset. Considering the differences in scanners, imaging parameters, and head sizes and orientations, five transformations (rotating: [−30°, 30°], shifting: [−0.1, 0.1], scaling: [0.95, 1.05], shearing: [0.1], and flipping) within the parameters range were applied for each slice in every epoch.

### U-net Architecture

The segmentation of WML and AIL was based on a U-net architecture described elsewhere (24). Each slice was fed into three U-net models as follows:

1. F model: each FLAIR image with the ground truth of WML was fed into the network as an input for WML segmentation;
2. Cascade model: each FLAIR image with the ground truth of WML was fed into the network as an input for WML segmentation, while each DWI image with the ground truth of AIL was fed into another network as an input for AIL segmentation;
3. FD model: the FLAIR and DWI images only with the ground truth of WML were fed into the network as a two-channels input for WML segmentation.

In Cascade model, the segmentation results were the regions identified as WML on FLAIR images but not identified as AIL on DWI images. All the networks were trained using a stochastic gradient descent (SGD) to minimize pixel-wise cross entropy. In order to compare the performance of different models in WML identification and segmentation, the epoch in each model training was set as 100. The initial learning rate was set to 0.01 and batch size was set to 4.

Among all 208 cases, 108 were randomly selected as training dataset and remaining 100 cases were used to evaluate the performance of segmentation. Table 3 summarizes subject demographics of training and testing set. There were no significant differences in sex, age, percentage of the subjects with AIL, height, weight and body mass index (all *p* > 0.05). In addition, 20 cases were randomly selected for testing the inter-observer reproducibility of the manual delineation of contour. Two observers independently delineated the WML.

**Table 3:** Comparison of clinic information between training and testing sets.

	**Training set**	**Testing set**	***P*-value**
	**(*n* = 108)**	**(*n* = 100)**	
Male/Female	82/26	74/26	0.749
Patients with AIL, %	48% (52)	46% (46)	0.756
Age, years	63.06 ± 12.08	64.94 ± 9.93	0.225
Height, cm	169.03 ± 6.97	168.16 ± 6.78	0.364
Weight, kg	68.61 ± 10.32	68.08 ± 9.54	0.701
BMI, kg/m^2^	23.96 ± 2.99	24.03 ± 2.75	0.865

*AIL, acute ischemic lesion; BMI, body mass index*.

### Post-processing

The predictive values ranging from 0 to 1 were obtained from U-net. Firstly, the pixels with the probability over 0.5 were seen as the candidates of WML. Secondly, the lesions which contained <5 pixels or were not in the white matter regions (probability <0.4 using SPM) were removed. Secondly, WML volume (WMLV) in each subject was calculated by the product of all lesion volumes and voxel volume. Finally, a rating equation was obtained through the linear regression between the ground truth and the scores in training set, and the scores in test set were calculated *via* substituting the measured WMLV into the rating equation.

Additionally, the Lesion Segmentation Toolbox (LST) (https://www.statistical-modelling.de/lst.html) was also used in segmentation of WML in the present study. LST has been validated as an efficient tool in detecting WML (25). In the present study, the performance in WML identification and segmentation was also compared between LST and U-net.

### Statistical Analysis

Spearman correlation coefficient and intraclass correlation coefficient (ICC) were calculated to evaluate the agreement between the score determined by observers and the score determined by U-net. For testing the reproducibility, ICC and corresponding 95% confidence interval (CI) were calculated to determine the inter-operator reproducibility of the manual delineation of contour. A *p*-value <0.05 was considered statistically significant, and all statistical analyses were conducted in SPSS 25.0 (IBM Inc., USA).

## Results

All continuous variables were presented as mean ± standard deviation (SD). In the present study, two categories were taken to compare the performance among different methods:

1. all subjects were divided into three subgroups according to WMLV: mild (WMLV ≤ 5 ml), moderate (5 ml < WMLV ≤ 20 ml), and severe (WMLV > 20 ml) (26).
2. all subjects were divided into two subgroups: the subjects with AIL (AILg) and those without AIL (non-AILg).

All the experiments were conducted on a RTX 2070 Super GPU with 8G memory and implemented on TensorFlow.

### Evaluation at Pixel Level

**Figure 2:**
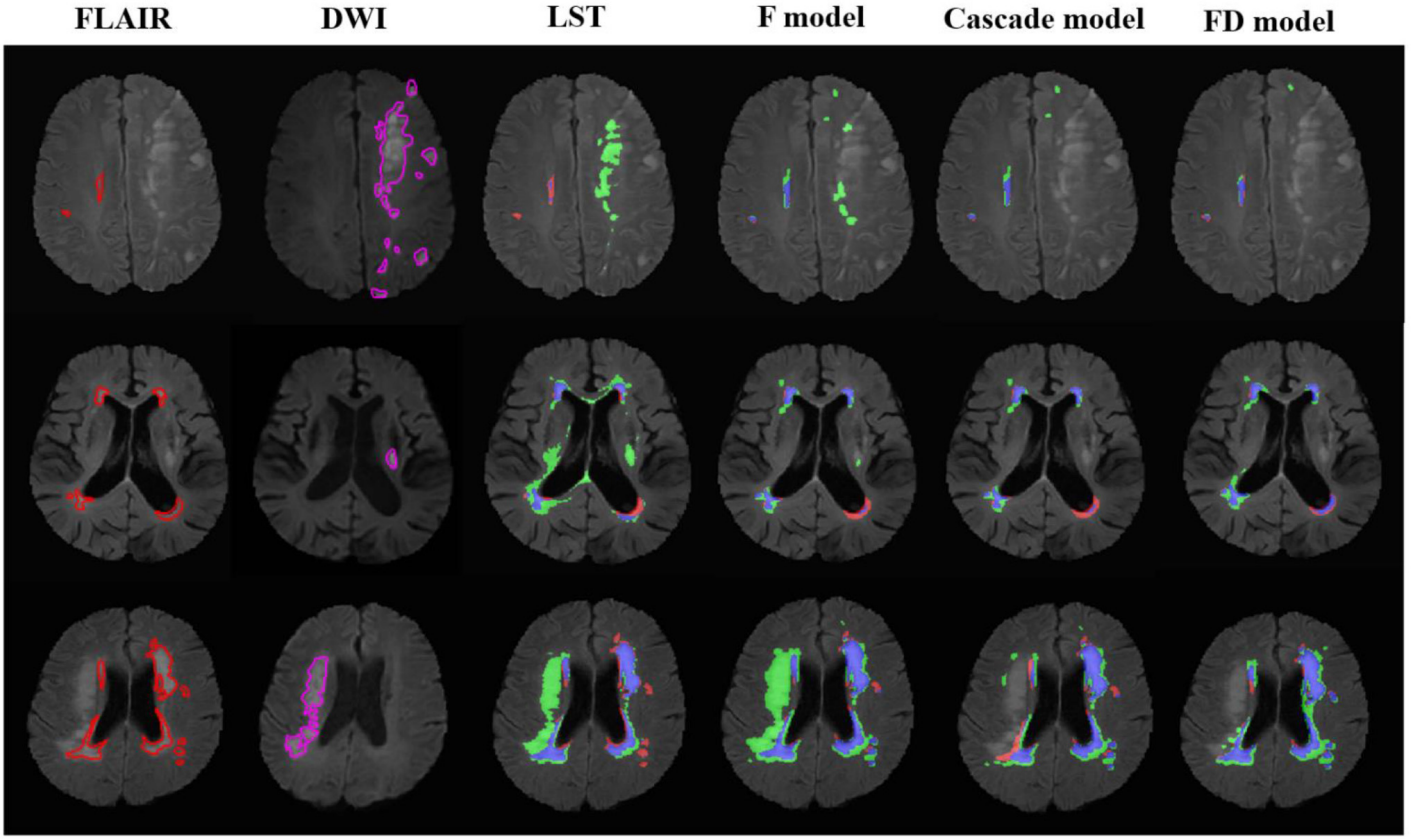
The performance of the LST and U-net models in the patients with acute ischemic lesions. The upper row was the case with white matter lesions volume (WMLV) ≤ 5 ml. The second row was the case with WMLV > 5 ml and WMLV ≤ 20 ml. The third row was the case with WMLV > 20 ml. The contours on FLAIR images were the ground truth of WML, and the contours on DWI images were the ground truth of AIL. On the images with segmented results by four methods, the red, green, and blue regions were the ground truth, segmented result and the overlap of the ground truth and segmented results, respectively.

Table 4 summarizes the performance of LST and U-net in WML segmentation at pixel level. Compared with LST, all U-net models exhibited higher DSC (LST: 0.39 ± 0.22, F model: 0.60 ± 0.14, Cascade model: 0.61 ± 0.13, and FD model: 0.61 ± 0.13), higher Recall (LST: 0.38 ± 0.23, F model: 0.73 ± 0.16, Cascade model: 0.72 ± 0.16, and FD model: 0.74 ± 0.18), and similar Precision (LST: 0.53 ± 0.26, F model: 0.54 ± 0.15, Cascade model: 0.56 ± 0.14, and FD model: 0.55 ± 0.13). All methods showed worse performance in DSC, Recall, and Precision in mild group, while exhibited better performance in DSC, Recall, and Precision in severe group (Figure 2). Additionally, LST exhibited similar performance between the cases with and without AIL. However, the performance of U-net in the cases without AIL was better than that in the cases with AIL.

**Table 4:** The performance of four segmentation methods at pixel level.

	**All**	**Category 1**			**Category 2**	
	**All (*n* = 100)**	**Mild (*n* = 43)**	**Moderate (*n* = 31)**	**Severe (*n* = 26)**	**AILg (*n* = 46)**	**Non-AILg (*n* = 54)**
**LST**						
DSC	0.39 ± 0.22	0.22 ± 0.14	0.44 ± 0.17	0.62 ± 0.11	0.38 ± 0.24	0.40 ± 0.20
Precision	0.53 ± 0.26	0.41 ± 0.28	0.53 ± 0.22	0.73 ± 0.13	0.47 ± 0.28	0.59 ± 0.24
Recall	0.38 ± 0.23	0.21 ± 0.17	0.44 ± 0.19	0.58 ± 0.15	0.38 ± 0.23	0.37 ± 0.23
**F MODEL**						
DSC	0.60 ± 0.14	0.50 ± 0.14	0.62 ± 0.09	0.72 ± 0.07	0.56 ± 0.17	0.63 ± 0.11
Precision	0.54 ± 0.15	0.45 ± 0.16	0.54 ± 0.10	0.66 ± 0.09	0.50 ± 0.17	0.56 ± 0.13
Recall	0.73 ± 0.16	0.66 ± 0.18	0.75 ± 0.13	0.81 ± 0.11	0.71 ± 0.20	0.74 ± 0.12
**CASCADE MODEL**						
DSC	0.61 ± 0.13	0.52 ± 0.13	0.64 ± 0.08	0.73 ±0.07	0.59 ± 0.15	0.63 ± 0.11
Precision	0.56 ± 0.14	0.48 ± 0.14	0.56 ± 0.09	0.67 ± 0.08	0.55 ± 0.15	0.56 ± 0.13
Recall	0.72 ± 0.16	0.65 ± 0.18	0.75 ± 0.13	0.81 ± 0.11	0.70 ± 0.19	0.74 ± 0.12
**FD MODEL**						
DSC	0.61 ±0.13	0.52 ± 0.13	0.63 ± 0.09	0.72 ± 0.06	0.59 ± 0.16	0.62 ± 0.11
Precision	0.55 ± 0.13	0.48 ± 0.14	0.55 ± 0.10	0.64 ± 0.08	0.54 ± 0.15	0.55 ± 0.13
Recall	0.74 ± 0.18	0.66 ± 0.20	0.76 ± 0.14	0.84 ± 0.09	0.72 ± 0.21	0.75 ± 0.15

*AILg, group with acute ischemic lesions; non-AILg, group without acute ischemic lesions; DSC, dice similarity coefficient*.

### Evaluation at Lesion Level

**Figure 3:**
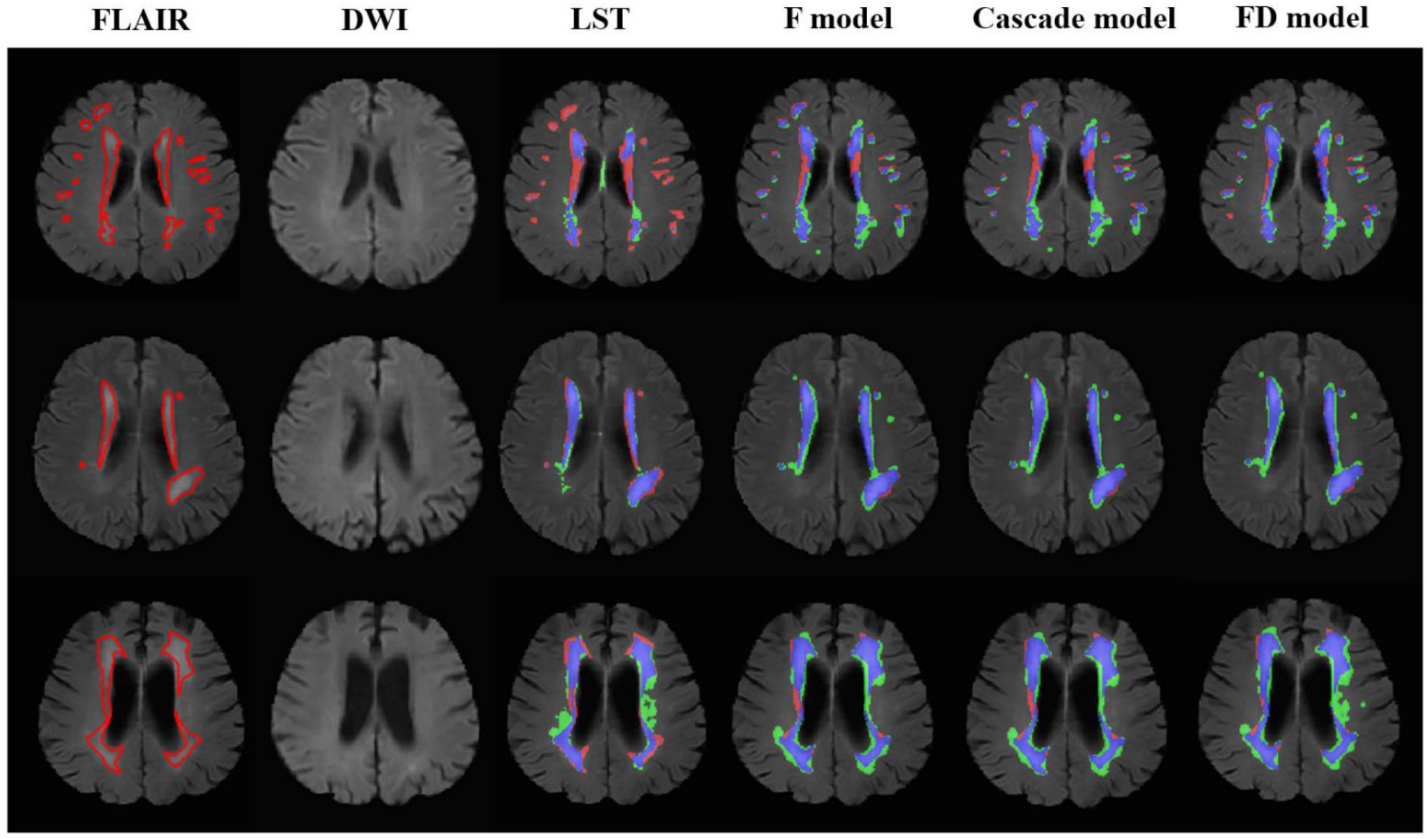
The performance of the LST and U-net models in the patients without acute ischemic lesions. The upper row was the case with white matter lesions volume (WMLV) ≤ 5 ml. The second row was the case with WMLV > 5 ml and WMLV ≤ 20 ml. The third row was the case with WMLV > 20 ml. On the images with segmented results by four methods, the red, green, and blue regions were the ground truth, segmented result and the overlap of the ground truth and segmented results, respectively.

The performance of LST and U-net at lesion level is shown in Table 5. Compared with LST, all U-net models exhibited higher TPR (LST: 0.64 ± 0.25, F model: 0.89 ± 0.15, Cascade model: 0.89 ± 0.15, and FD model: 0.89 ± 0.15), and lower FPR (LST: 0.46 ± 0.21, F model: 0.34 ± 0.17, Cascade model: 0.34 ± 0.16, and FD model: 0.36 ± 0.15). Similar to the evaluation at pixel level, the performance of each method was improved from mild to moderate and then to severe (Figure 3). No difference in TPRs of LST and three U-net models was found between the cases with and without AIL, but all methods showed decreased FPRs in the cases without AIL compared with the cases with AIL.

**Table 5:** The performance of four segmentation methods at lesion level.

	**All**	**Category 1**			**Category 2**	
	**All (*n* = 100)**	**Mild (*n* = 43)**	**Moderate (*n* = 31)**	**Severe (*n* = 26)**	**AILg (*n* = 46)**	**Non-AILg (*n* = 54)**
**LST**						
TPR	0.64 ± 0.25	0.59 ± 0.30	0.67 ± 0.20	0.71 ± 0.20	0.65 ± 0.25	0.64 ± 0.26
FPR	0.46 ± 0.21	0.52 ± 0.25	0.48 ± 0.16	0.34 ± 0.13	0.50 ± 0.21	0.43 ± 0.21
**F MODEL**						
TPR	0.89 ± 0.15	0.86 ± 0.20	0.89 ± 0.12	0.93 ± 0.05	0.87 ± 0.20	0.91 ± 0.09
FPR	0.34 ± 0.17	0.40 ± 0.20	0.33 ± 0.14	0.26 ± 0.11	0.40 ± 0.18	0.29 ± 0.15
**CASCADE MODEL**						
TPR	0.89 ± 0.15	0.86 ± 0.20	0.89 ± 0.12	0.93 ± 0.05	0.87 ± 0.20	0.91 ± 0.09
FPR	0.34 ± 0.16	0.39 ± 0.19	0.33 ± 0.14	0.26 ± 0.11	0.39 ± 0.17	0.29 ± 0.15
**FD MODEL**						
TPR	0.89 ± 0.15	0.87 ± 0.20	0.88 ± 0.11	0.95 ±0.05	0.88 ± 0.19	0.91 ± 0.09
FPR	0.36 ± 0.15	0.41 ± 0.16	0.35 ± 0.15	0.30 ± 0.13	0.39 ± 0.14	0.34 ± 0.16

*AILg, group with acute ischemic lesions; Non-AILg, group without acute ischemic lesions; TPR, true positive rate; FPR, false positive rate*.

### Evaluation on Subject Level

**Figure 4:**
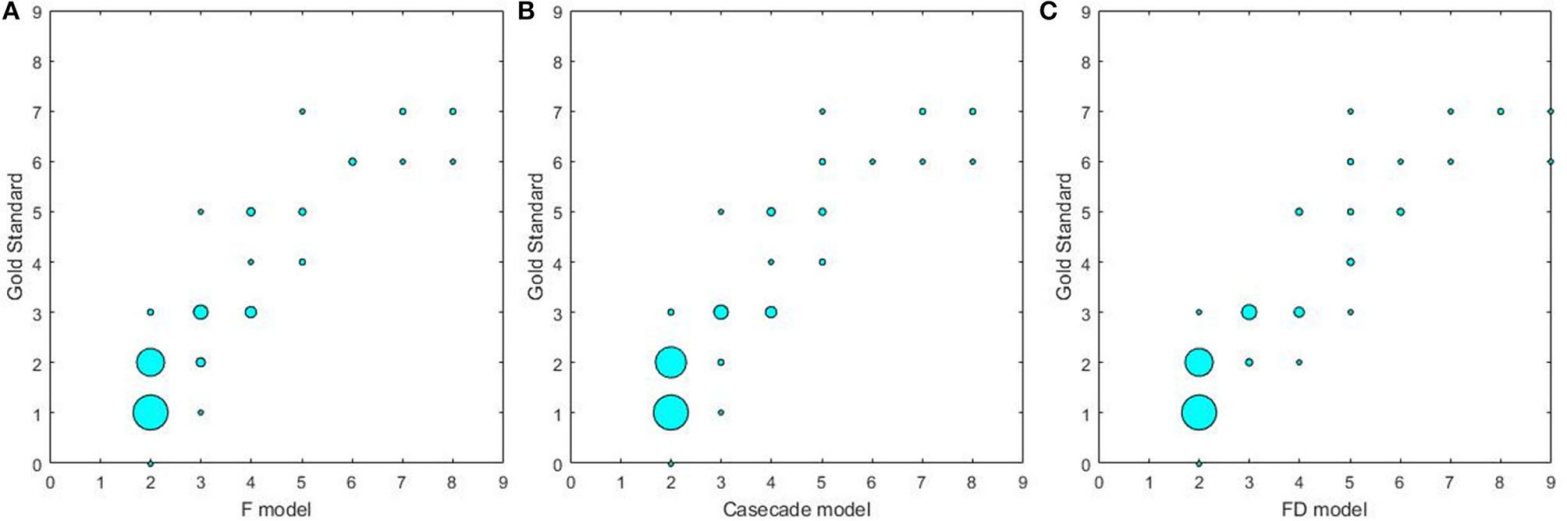
The correlations between different U-net models and gold standard. **(A)** The correlation between F model and gold standard; **(B)** the correlation between Cascade model and gold standard; **(C)** the correlation between FD model and gold standard.

The results on the correlations between the scores determined by the radiologists and by three U-net models are summarized in Table 6 and Figure 4. Each score evaluated by three U-net models exhibited a strong correlation with the score determined by the radiologists (all *r* > 0.85, *p* < 0.001, and ICC > 0.86). The correlations of the scores determined by three U-net models were also compared, and no significant differences were found in WML scores among three U-net models (all *p* > 0.05).

**Table 6:** The correlation between scores offered by radiologist and by U-net.

		**F model**	**Cascade model**	**FD model**
All	SC	*r* = 0.856, *p* < 0.001	*r* = 0.857, *p* < 0.001	*r* = 0.881, *p* < 0.001
(*n* = 100)	ICC	0.872, 95% CI: 0.723–0.932	0.871, 95% CI: 0.757–0.926	0.867, 95% CI: 0.649–0.935
AILg	SC	*r* = 0.873, *p* < 0.001	*r* = 0.885, *p* < 0.001	*r* = 0.858, *p* < 0.001
(*n* = 46)	ICC	0.883, 95% CI: 0.493–0.957	0.883, 95% CI: 0.678–0.947	0.841, 95% CI: 0.540–0.931
Non-AILg	SC	*r* = 0.903, *p* < 0.001	*r* = 0.903, *p* < 0.001	*r* = 0.928, *p* < 0.001
(*n* = 54)	ICC	0.863, 95% CI: 0.757–0.922	0.863, 95% CI: 0.757–0.922	0.891, 95% CI: 0.714–0.950

*SC, Spearman coefficient; ICC, interclass correlation coefficient; CI, confidence interval. AILg, the group with acute ischemic lesions; Non-AILg, the group without acute ischemic lesions*.

### Reproducibility of Manual Lesion Identification and Segmentation

The DSC of the contours manually delineated by two observers was 0.65 ± 0.06. The inter-observer ICCs of WMLV and score were 0.991 (95% CI: 0.976–0.997) and 0.792 (95% CI: 0.357–0.926), respectively.

## Discussion

In the present study, we used the U-net architecture to identify and segment WML in the patients with both WML and AIL, and compared the performance of three models. The results of three models and LST were compared at pixel, lesion, and subject levels. Three U-net models all outperformed LST at each metrics *via* testing on the dataset from a multi-center study. However, the introduction of DWI sequence in U-net didn't significantly improve the identification and segmentation of WML in the cases with AIL.

Compared with LST, U-net exhibited a significant advantage in detecting WML both in the cases with and without AIL. Though CNN has been validated as a powerful tool in classification, identification and segmentation, it is limited in some fields due to the output variance across different training sessions. Many studies had validated that CNN-based architectures could obtain better performance in WML segmentation by combining FLAIR and T1W images (13, 15). However, seldom research investigated the patients suffered from both WML and AIL. The similar intensity between AIL and WML on FLAIR images limited the performance of CNN architecture in WML evaluation only with FLAIR images. No significant difference in identification and segmentation of lesions between F and FD models was found in the present study. This might imply that the CNN-based architectures could distinguish WML from AIL on FLAIR images through some features.

Our study found that LST and all U-net models exhibited best performance in severe group. In mild group, it is a tough work for reviewers to recognize and delineate the contour of the lesions. Jose et al. (27) proposed an ensemble of neural networks and overcomplete patch-based voting to automatically segment WML. Our results showed similar phenomenon in WML segmentation. All methods performed better for the lesions with larger size. The lesions with large size were easy to detect, and the evaluation metrics were less affected by the mismatch along the boundary. Additionally, FD model exhibited a slight increase in TPR compared with F and Cascade models, while its FPR was also higher than the FPRs of F and Cascade models. This may indicate that the improvement in segmentation of WML by combining FLAIR and DWI was accompanied with the increase in misidentifying WML.

LST and three U-net models exhibited similar TPRs between AIL and non-AIL group, but decreased FPRs in non-AIL group. This favored the profile of similar signal intensity between infarction and WML on FLAIR images. DWI sequence is a MR imaging technique to detect the infarction in brain. However, it is interesting that all three U-net models exhibited similar performance in both AIL group and non-AIL group. This suggests that DWI contributed less to WML identification and segmentation in U-net.

The strong correlation between the scores determined by U-net models and the gold standard suggests that neural network might be an alternative for automatic WML rating in the subjects with WML and AIL. However, this study suffered from several limitations. First, T1 sequence was missing and only a single U-net was used in the current study. Complementary information obtained from T1, DWI and FLAIR images could improve the performance of U-net in identification and segmentation of WML, while multiple U-net models could reduce the over-fitting problems on data training (28). Second, the common limitation in WML segmentation is the lack of accepted reference. Though the contour delineated by experienced reviewers could be a surrogate for the gold standard, this evaluation is still subjective, especially in rating the WML. WMLV and rating might not be a robust index to evaluate the performance of different methods. The associations between the segmented results and clinical events should be investigated. Finally, only twenty cases were used to test the reproducibility of manual delineation.

## Conclusion

U-net performs well in identification and segmentation of WML in the patients with and without AIL on FLAIR and DWI images. The U-net only using FLAIR images exhibits similar performance in WML identification and segmentation with those using the combination of DWI and FLAIR images. Our study was validated by a dataset of multicenter study and indicates that U-net has an advantage in assessing burden of WML in the patients suffered from both WML and AIL.

## Data Availability Statement

SL and XZ are responsible for all datasets presented in the article, which will be made available to any qualified researcher upon request.

## Ethics Statement

The studies involving human participants were reviewed and approved by Institutional Review Board of Tsinghua University School of Medicine. The patients/participants provided their written informed consent to participate in this study.

## Author Contributions

SL, FS, and XZ conceived and designed the study. SL, XW, SH, and FS involved in the MR data analysis. SL, XS, SH, and XW performed the statistical analysis. SL and FS drafted the manuscript. XS and XZ revised the manuscript. All authors contributed to the article and approved the submitted version.

## Conflict of Interest

The authors declare that the research was conducted in the absence of any commercial or financial relationships that could be construed as a potential conflict of interest.
